# Supplementary material for: Microfluidic Array Enables Rapid Testing of Natural Compounds Against Xylella fastidiosa
Source: Plants (Basel). 2025 Mar 11;14(6):872. doi: 10.3390/plants14060872 (PMC11946115; doi:10.3390/plants14060872)
Supplement: Supplementary file 1 [file plants-14-00872-s001.zip › plants-3489935-supplementary.pdf]

Supplementary Materials

Microfluidic Array Enables Rapid Testing of Natural Compounds Against *Xylella fastidiosa*

Francesca Costantini [1,*,†], Erica Cesari [1,†], Nicola Lovecchio [2], Marco Scortichini [3], Valeria Scala [1], Stefania Loreti [1] and Nicoletta Pucci [1]

[1] Research Centre for Plant Protection and Certification, Council for Agricultural Research and Economics (CREA-DC), 00156 Rome, Italy; erica.cesari@crea.gov.it (E.C.); valeria.scala@crea.gov.it (V.S.); stefania.loreti@crea.gov.it (S.L.); nicoletta.pucci@crea.gov.it (N.P.)
[2] Department of Information Engineering, Electronics and Telecommunications, Sapienza University of Rome, 00184 Rome, Italy; nicola.lovecchio@uniroma1.it
[3] Research Centre for Olive, Fruit and Citrus Crops, Council for Agricultural Research and Economics (CREA-OFA), 00134 Rome, Italy; mscortichini@yahoo.it
* Correspondence: francesca.costantini@crea.gov.it
† These authors contributed equally to this work.

S1 Fabrication of the microfluidic channel array

The PDMS slab was fabricated following a previously reported procedure (Costanni et al., 2016; Mirasoli et al., 2018) and bonded to the glass slide using the transfer bonding technique with uncured PDMS as the adhesive. Briefly 1–2 mL of PDMS mixture 10 (base material): 1 (curing agent) ratio was poured on a glass slide (5 × 5 cm^2) 1 mm thickness, which was then spin-coated at 4500 rpm for 4 min (Spincoater laurel WS-650-23). This resulted in a thin layer of uncured PDMS. The previously fabricated PDMS slab was placed on this glass slide and left for 1–2 min. Then, the PDMS cured slab was lifted off, leaving a layer of uncured PDMS on it and placed on a glass slide having the 0.2 mm thickness, previously cleaned by oxygen plasma (Diener Zepto plasma cleaner, Germany) for 10 min (100 W) and cured at 90 °C for 15 min. The inlet and outlet connections were made by inserting stainless steel pins (0.013″ ID × 0.025″ OD × 0.5″ long) purchased by New England Small Tube Corporation (Litchfield, NH, USA) in the inlet/outlet hole previously formed. Solutions were flowed in/out of the microfluidic channels by connecting tygon tubing (0.020″ ID × 0.060″ OD) purchased by General control S.p.A. (Milan, Italy) to the pins inserted in the device. Sample solutions were flowed pressure-driven into the microfluidic chip by means of a PHD 2000 series syringe pump (Harvard Apparatus, Crisel Instruments S.r.l, Rome, Italy) equipped with six sterile plastic syringes of 1 mL volume (Therumo), having needles of the following dimensions: 0.8 X 38 mm.

Prior use, the device was kept under UV light for 10 min and rinsed out for 1h with ethanol (70%) at the flow-rate of 0.1 µL/ min for 10 min and rinsed out with sterilized MilliQ water.

Academic Editor: Fabrício Ávila Rodrigues

Received: 6 February 2025
Revised: 3 March 2025
Accepted: 6 March 2025
Published: date

Citation: Costantini, F.; Cesari, E.; Lovecchio, N.; Scortichini, M.; Scala, V.; Loreti, S.; Pucci, N. Microfluidic Array Enables Rapid Testing of Natural Compounds Against *Xylella fastidiosa*. *Plants* **2025**, *14*, x. https://doi.org/10.3390/xxxxx

Copyright: © 2025 by the authors. Submitted for possible open access publication under the terms and conditions of the Creative Commons Attribution (CC BY) license (https://creativecommons.org/licenses/by/4.0/).

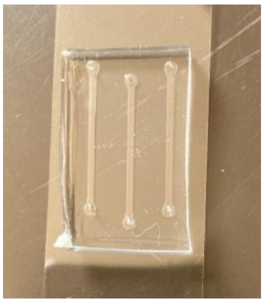

Figure S1: Picture of the glass-PDMS microfluidic chip.

S2 Comparison of *Xylella fastidiosa* subsp. *fastidiosa* Growth in Glass Tube versus Microfluidic Channels

The *Xylella fastidiosa* subsp. *fastidiosa* (*Xff*) colony increment ratio (R) can be used to compare the grow rate of the bacteria using the following equations:

a. Bacterial suspension in glass tube: R= OD/OD_0, where OD_0 and OD are the optical density (OD_{600}) of suspended *Xff* cells initially (t=0) and after a time t= 3 and 6 days.
b. Bacterial suspension in microfluidic channel: R= S/S_0, where S_0 and S are *Xff* colony areas initially (t=0) and after a time t= 3 and 6 days.

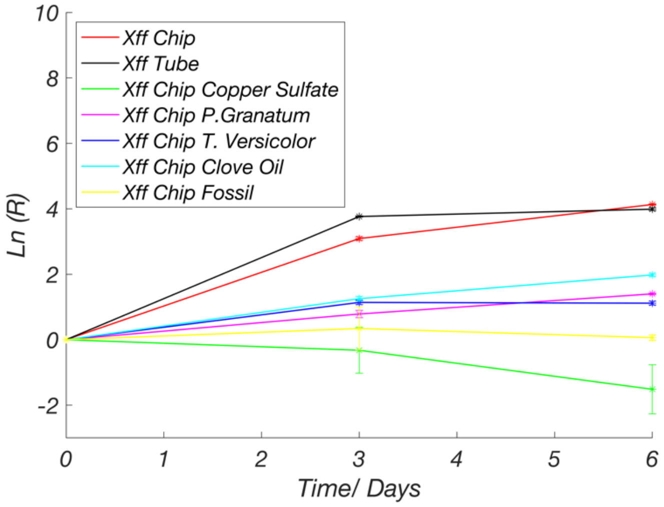

Figure S2: Comparison of the increment ratio (R) of *Xylella fastidiosa* subsp. *fastidiosa* (*Xff*) colonies growing in glass tube (*Xff* Tube) or microfluidic channels (*Xff* Chip) as function of time.
